# Trimethoprim-sulfamethoxazole dosing and outcomes of pulmonary nocardiosis

**DOI:** 10.1007/s15010-024-02323-9

**Published:** 2024-06-26

**Authors:** Zachary A. Yetmar, Ryan B. Khodadadi, Supavit Chesdachai, Jack W. McHugh, Josh Clement, Douglas W. Challener, Nancy L. Wengenack, Wendelyn Bosch, Maria Teresa Seville, Elena Beam

**Affiliations:** 1 Division of Public Health, Infectious Diseases, and Occupational Medicine, Mayo Clinic, 200 First Street SW, Rochester, MN 55905 USA; 2 Department of Infectious Disease, Cleveland Clinic Foundation, Cleveland, OH USA; 3 Department of Pharmacy, Mayo Clinic, Rochester, MN USA; 4 Division of Clinical Microbiology, Mayo Clinic, Rochester, MN USA; 5 Division of Infectious Diseases, Mayo Clinic, Jacksonville, FL USA; 6 Division of Infectious Diseases, Mayo Clinic, Phoenix, Arizona USA

**Keywords:** *Nocardia*, Bactrim, Bronchiectasis, Sulfonamide, Dose

## Abstract

**Background:**

*Nocardia* often causes pulmonary infection among those with chronic pulmonary disease or immunocompromising conditions. Trimethoprim-sulfamethoxazole (TMP-SMX) is recommended as first-line treatment, though little data exists regarding outcomes of different dosing regimens.

**Methods:**

We performed a multicenter retrospective cohort study of adult patients with non-disseminated pulmonary nocardiosis initially treated with TMP-SMX monotherapy. Patients’ initial TMP-SMX dosing was categorized as high- (> 10 mg/kg/day), intermediate- (5–10 mg/kg/day) or low-dose (< 5 mg/kg/day). Outcomes included one-year mortality, post-treatment recurrence, and dose adjustment or early discontinuation of TMP-SMX. SMX serum concentrations and their effect on management were also assessed. Inverse probability of treatment weighting was applied to Cox regression analyses.

**Results:**

Ninety-one patients were included with 24 (26.4%), 37 (40.7%), and 30 (33.0%) treated with high-, intermediate-, and low-dose TMP-SMX, respectively. Patients who initially received low-dose (HR 0.07, 95% CI 0.01–0.68) and intermediate-dose TMP-SMX (HR 0.27, 95% CI 0.07–1.04) had lower risk of one-year mortality than the high-dose group. Risk of recurrence was similar between groups. Nineteen patients had peak SMX serum concentrations measured which resulted in 7 (36.8%) dose changes and was not associated with one-year mortality or recurrence. However, 66.7% of the high-dose group required TMP-SMX dose adjustment/discontinuation compared to 24.3% of the intermediate-dose and 26.7% of the low-dose groups (*p* = 0.001).

**Conclusions:**

Low- and intermediate-dose TMP-SMX for non-disseminated pulmonary nocardiosis were not associated with poor outcomes compared to high-dose therapy, which had a higher rate of dose adjustment/early discontinuation. Historically used high-dose TMP-SMX may not be necessary for management of isolated pulmonary nocardiosis.

## Background

*Nocardia* is a saprophytic, partially acid-fast pathogen that primarily causes pulmonary disease due to environmental inhalation. Infection from this bacterium is estimated to occur in up to 0.87 per 100,000 people annually [1, 2]. Approximately 80% of cases involve the lungs, with most being non-disseminated at presentation [3, 4]. The most affected groups are individuals with chronic pulmonary disease or immunocompromising conditions.

Historically, first-line treatment for nocardiosis has been trimethoprim-sulfamethoxazole (TMP-SMX) [5]. This is often given as monotherapy, though patients with severe infection may receive a combination of multiple agents. Limited data have suggested patients who receive TMP-SMX have better outcomes than those who receive alternative therapies [6]. In view of this, TMP-SMX has been recommended as initial treatment, typically at a dose of 15 mg/kg of TMP per day [7]. However, this dosing regimen is extrapolated from treatment for *Pneumocystis* pneumonia, and more recent studies have questioned the need for such high-dose treatment, even for *Pneumocystis* [8]. Also extrapolated from *Pneumocystis* treatment is a goal peak SMX concentration of 100–150 µg/mL, though there is little data examining this practice among patients with nocardiosis and studies have not definitively found differences in outcomes based on peak SMX concentrations [9, 10].

In this study, we aimed to evaluate the outcomes of non-disseminated pulmonary nocardiosis based on initial TMP-SMX dosing, hypothesizing that patients would have similar outcomes despite initial TMP-SMX dosing. We also assessed the rates of dose adjustment or early discontinuation due to adverse effects attributed to TMP-SMX, as well as the use of SMX serum concentrations and their effect on clinical decision making.

## Methods

### Study design

We performed a retrospective cohort study of adult patients with non-disseminated pulmonary nocardiosis whose initial treatment was TMP-SMX monotherapy at three Mayo Clinic hospitals in Arizona, Florida, and Minnesota. Patients were diagnosed between November 2011 and April 2022 and were included in a prior study [3]. Patients were obtained through microbiology culture records and screened for inclusion through pre-determined criteria. Inclusion criteria were age ≥ 18 years on the date of diagnosis, culture growth of a *Nocardia* species with compatible signs, symptoms, and/or radiographic features consistent with infection, pulmonary involvement of nocardiosis, TMP-SMX susceptibility confirmed by in vitro antimicrobial susceptibility testing, and initial TMP-SMX monotherapy. Exclusion criteria were lack of culture-confirmation of nocardiosis, initial combination or non-TMP-SMX therapy, disseminated infection (involvement of at least 2 non-contiguous anatomic sites), TMP-SMX non-susceptibility or lack of valid susceptibility results, and lack of research authorization per Minnesota state statute. For patients who experienced multiple episodes of nocardiosis, only the first episode was included. Once cases were screened for inclusion, data were manually extracted from the electronic medical record. Study data were collected and managed using REDCap electronic data capture tools [11, 12].

### SMX serum concentrations

SMX serum concentrations were measured by liquid-chromatography mass spectrometry at our institution’s Clinical Toxicology and Drug Monitoring Laboratory [13]. This is typically drawn as a peak concentration 1 h after the last intravenous dose or 2 h after the last oral dose of TMP-SMX. When assessed, our standard practice is to target a peak SMX serum concentration of 100–150 µg/mL. This testing was available to patients at all included sites.

### Identification and susceptibility testing

The Clinical Microbiology laboratory at Mayo Clinic in Rochester, Minnesota, received specimens for culture, identification, and susceptibility testing from all included Mayo Clinic sites. Clinical specimens were cultured in BD BACTEC Mycobacterial Growth Indicator Tube 960 broth in Mycobacterial Growth Indicator Tubes (MGIT; Becton, Dickinson and Company, Franklin Lakes, NJ, USA) and on Middlebrook 7H11/7H11S agar biplates incubated at 35^o^C to 37^o^C for up to 6 weeks. Positive MGIT broth was subcultured to a Middlebrook 7H11 agar plate and isolated colony growth was originally identified using Sanger sequencing of a 500-bp region of the 16 S rRNA gene as previously described [14]. From August 2014, matrix-assisted laser desorption ionization time-of-flight mass spectrophotometry was introduced for species identification, with Sanger sequencing being reserved for isolates unable to be identified by this technique [15, 16]. Isolates that were unable to be identified to the species level were assumed to not be *N. farcinica*, as the standard identification methods should reliably identify this species [15–18]. Antimicrobial susceptibility testing was performed via broth microdilution using the Trek Sensititre® RAPMYCO plate (RAPMYCO; Thermo Scientific, Cleveland, OH, USA) and interpreted according to the Clinical and Laboratory Standards Institute guidelines during the respective period [19, 20]. Species identification and antimicrobial susceptibility testing was routinely attempted for all *Nocardia* isolates.

### Statistical analysis

Continuous variables were summarized as median (interquartile range [IQR]) and categorical variables as number (percentage). Patients were categorized into high, intermediate, and low treatment groups if the initial daily trimethoprim dose was > 10 mg/kg, 5–10 mg/kg, and < 5 mg/kg, respectively, after accounting for kidney function. TMP-SMX doses were adjusted with a correction factor to account for drug exposure based on creatinine clearance (incorporating adjusted body weight for patients whose weight was > 120% of ideal body weight [21, 22]). Specifically, TMP-SMX doses of patients with creatinine clearance < 15 mL/min were multiplied by 3, and doses of patients with creatinine clearance 15–30 mL/min were multiplied by 2 [23]. The primary outcome was 1-year all-cause mortality. The secondary outcomes were all-time *Nocardia* recurrence and a composite of dose adjustment or early discontinuation of TMP-SMX. Recurrence was defined as culture growth of a *Nocardia* species with accompanying signs, symptoms, and/or radiographic features consistent with infection any time after completion of primary *Nocardia* therapy. For the recurrence analyses, patients were excluded if they had not completed primary therapy due to either death or loss to follow-up. Kaplan-Meier curves were constructed to illustrate differences in cumulative incidence of the outcomes after the respective index dates. Differences in outcomes between groups were tested by the log-rank test. Cox proportional hazards regression was used to analyze associations with the primary outcome. To account for potential imbalances between treatment groups, a propensity score was constructed by multinomial regression and applied using inverse probability of treatment weighting. The propensity score was defined *a priori* and included sex, age, chronic pulmonary disease, Charlson comorbidity index, chronic kidney disease (baseline estimated glomerular filtration rate < 60 mL/min/1.73 m^2^, calculated by the 2021 CKD-EPI Eq. [24]), solid organ transplantation, hematopoietic stem cell transplantation, immunosuppressant use within the last 28 days, infection with *N. farcinica*, hospitalization for nocardiosis, and pulmonary cavitation. Standardized mean difference (SMD), a sample size-independent value that estimates the difference of a variable between groups, was used to determine adequacy of balance after applying the weights [25]. For the purposes of this analysis, SMD ≤ 0.20 was considered acceptable balance. The Cox regression analyses were also stratified by treatment center. Finally, a pre-planned sensitivity analysis where TMP-SMX dosing were categorized as a binary variable using a breakpoint of 10 mg/kg/day (with refitting of the propensity score model for this variable), and subgroup analyses of the chronic pulmonary disease and immunocompromising medication use groups were performed. All analyses were performed using R version 4.3.1 (R Foundation for Statistical Computing, Vienna, Austria).

## Results

### Cohort characteristics

**Fig. 1:**
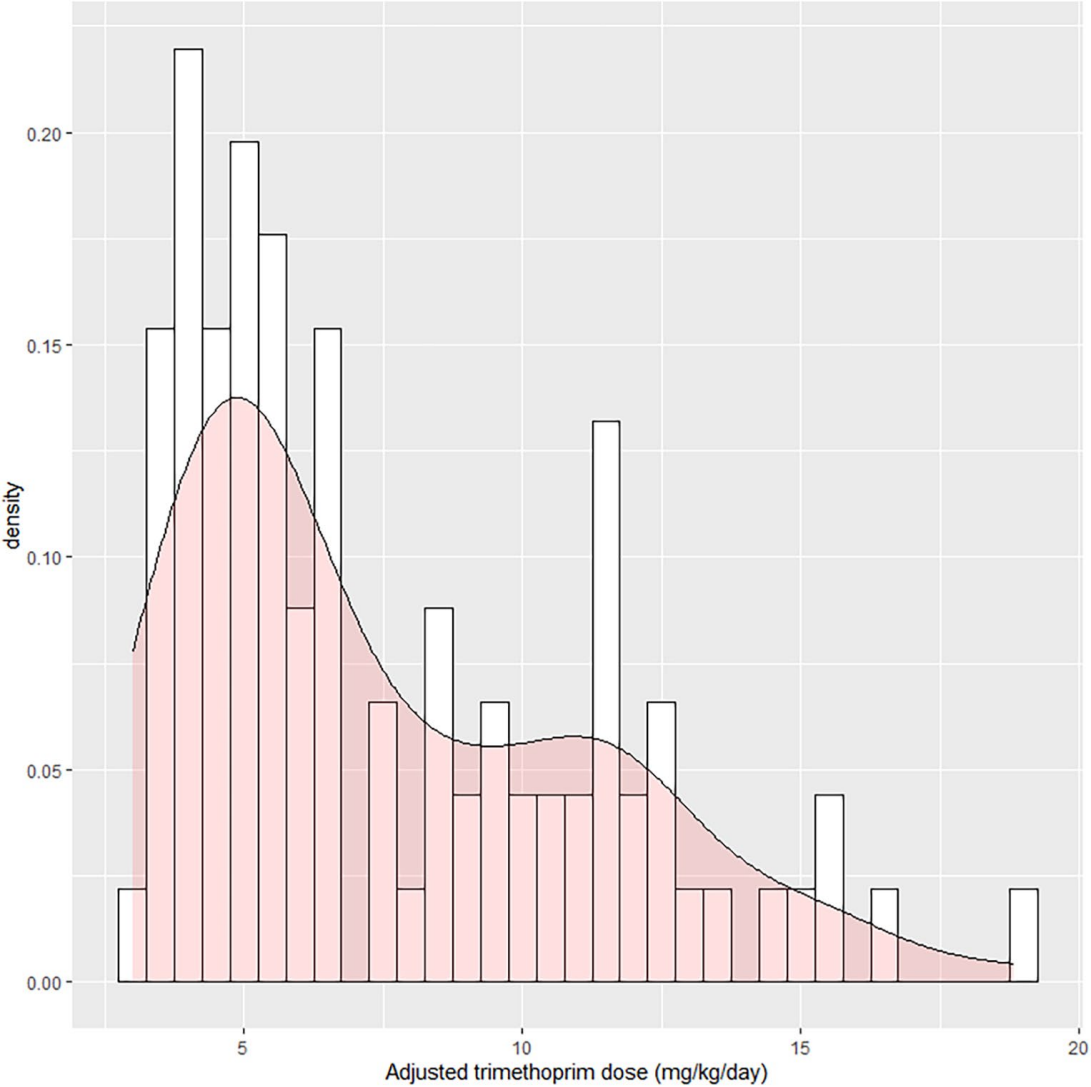
Histogram and density plot of initial, weight-based trimethoprim doses after adjusting for baseline creatinine clearance. The bars represent the number of patients with specific trimethoprim-sulfamethoxazole doses, with a bin width of 0.5 mg/kg/day of trimethoprim

From 243 patients with non-disseminated pulmonary nocardiosis, 8 were excluded due to not having susceptibility testing results available, 9 were excluded due to their *Nocardia* isolate being TMP-SMX non-susceptible, and 135 were excluded due to receiving initial therapy other than TMP-SMX monotherapy. This left 91 patients with non-disseminated pulmonary nocardiosis who were included in this study, of which 24 (26.4%) received high-dose, 37 (40.7%) intermediate-dose, and 30 (33.0%) low-dose initial TMP-SMX therapy (Fig. 1). Two patients had a creatinine clearance 15–30 mL/min and required dose adjustment. No patients’ baseline creatinine clearance was less than 15 mL/min. The median age was 70.5 years (IQR 62.2, 79.6). Four (4.4%) patients initially received intravenous treatment, and all were later transitioned to oral therapy. Most patients had chronic lung disease (*N* = 64, 70.3%), the most common etiology being non-cystic fibrosis bronchiectasis. Thirty-one patients (34.1%) were receiving immunosuppressing medications, with 9 (9.9%) having a prior solid organ transplant, 5 (5.5%) having received a hematopoietic stem cell transplant (4 allogeneic and 1 autologous), and 11 (12.1%) had an active malignancy. The high-dose TMP-SMX therapy group had a lower rate of chronic lung disease (50.0% versus 78.4% in the intermediate-dose group and 76.7% in the low-dose group), and higher rates of immunosuppressant use and immunocompromising conditions. The low-dose group had a higher weight compared to both the high- and intermediate-dose groups. The high- and intermediate-dose groups had similar rates of hospitalization of 33.3% and 27.0%, respectively, while the low-dose group had a lower rate of 13.3%. Only one patient (who received high-dose TMP-SMX) required intensive care unit admission. Forty-six patients (50.5%) underwent brain imaging, with the highest rate in the high-dose group. The most common *Nocardia* species were *N. cyriacigeorgica* (*N* = 28, 30.8%), *N. nova* (*N* = 19, 20.9%), *N. farcinica* (*N* = 17, 18.7%), and *N. wallacei* (*N* = 7, 7.7%). Thirteen patients received secondary prophylaxis after completing primary treatment, all with TMP-SMX. Secondary TMP-SMX prophylaxis was dosed as 160–800 mg twice-daily (*N* = 2/13), 160–800 daily (*N* = 3/13), 160–800 thrice-weekly (*N* = 3/13), 160–800 weekly (*N* = 2/13), 80–400 daily (*N* = 2/13), and 80–400 thrice-weekly (*N* = 1/13). Other cohort characteristics are displayed in Table 1.

**Table 1:** Cohort characteristics, grouped by trimethoprim dosing category

Characteristics	High (*N* = 24)	Intermediate (*N* = 37)	Low (*N* = 30)	Total (*N* = 91)
Age, years	71.0 (58.9, 76.7)	69.7 (62.5, 80.9)	71.8 (65.3, 76.2)	70.5 (62.2, 79.6)
**Sex**				
Female	14 (58.3%)	23 (62.2%)	12 (40.0%)	49 (53.8%)
Male	10 (41.7%)	14 (37.8%)	18 (60.0%)	42 (46.2%)
Weight, kg	60.0 (54.9, 78.6)	62.1 (55.0, 73.5)	78.8 (69.9, 85.4)	69.2 (57.5, 80.7)
Body mass index, kg/m^2^	24.2 (21.0, 27.9)	23.8 (21.0, 26.5)	27.9 (25.4, 31.5)	25.3 (22.2, 29.3)
Charlson comorbidity index	2.0 (1.0, 3.2)	2.0 (1.0, 3.0)	2.0 (1.0, 3.0)	2.0 (1.0, 3.0)
Chronic pulmonary disease	12 (50.0%)	29 (78.4%)	23 (76.7%)	64 (70.3%)
Asthma	1 (8.3%)	2 (6.9%)	0 (0.0%)	3 (4.7%)
Bronchiectasis	8 (66.7%)	21 (72.4%)	14 (60.9%)	43 (67.2%)
COPD	3 (25.0%)	3 (10.3%)	7 (30.4%)	13 (20.3%)
Cystic fibrosis	0 (0.0%)	0 (0.0%)	1 (4.3%)	1 (1.6%)
Interstitial lung disease	0 (0.0%)	3 (10.3%)	1 (4.3%)	4 (6.2%)
Diabetes mellitus	5 (20.8%)	3 (8.1%)	7 (23.3%)	15 (16.5%)
Solid organ transplant	3 (12.5%)	2 (5.4%)	4 (13.3%)	9 (9.9%)
Hematopoietic stem cell transplant	3 (12.5%)	1 (2.7%)	1 (3.3%)	5 (5.5%)
Active malignancy	4 (16.7%)	4 (10.8%)	3 (10.0%)	11 (12.1%)
Primary TMP-SMX prophylaxis	1 (4.2%)	2 (5.4%)	3 (10.0%)	6 (6.6%)
Immunosuppressant use within 28 days	12 (50.0%)	9 (24.3%)	10 (33.3%)	31 (34.1%)
Tacrolimus	4 (16.7%)	2 (5.4%)	4 (13.3%)	10 (11.0%)
Sirolimus	1 (4.2%)	0 (0.0%)	0 (0.0%)	1 (1.1%)
Mycophenolate	3 (12.5%)	1 (2.7%)	3 (10.0%)	7 (7.7%)
Azathioprine	0 (0.0%)	1 (2.7%)	0 (0.0%)	1 (1.1%)
Ruxolitinib	1 (4.2%)	1 (2.7%)	0 (0.0%)	2 (2.2%)
Methotrexate	3 (12.5%)	1 (2.7%)	0 (0.0%)	4 (4.4%)
Anti-CD20	1 (4.2%)	1 (2.7%)	1 (3.3%)	3 (3.3%)
Chemotherapy	2 (8.3%)	1 (2.7%)	1 (3.3%)	4 (4.4%)
Corticosteroid	9 (37.5%)	8 (21.6%)	8 (26.7%)	25 (27.5%)
Prednisone dose, mg	10.0 (5.0, 20.0)	13.8 (10.0, 22.5)	13.8 (10.0, 22.5)	10.0 (10.0, 20.0)
Brain imaging	18 (75.0%)	19 (51.4%)	9 (30.0%)	46 (50.5%)
CT	8 (33.3%)	10 (27.0%)	6 (20.0%)	24 (26.4%)
MRI	13 (54.2%)	10 (27.0%)	3 (10.0%)	26 (28.6%)
Leukocyte count, x10^9^/L	8.0 (5.8, 10.1)	8.3 (6.5, 10.7)	8.4 (6.3, 9.4)	8.1 (6.1, 10.1)
Neutrophil count, x10^9^/L	5.5 (3.4, 9.1)	5.6 (4.2, 7.4)	5.0 (3.5, 7.6)	5.4 (3.7, 7.6)
Lymphocyte count, x10^9^/L	1.0 (0.5, 1.5)	1.4 (0.9, 1.8)	1.3 (0.9, 2.0)	1.2 (0.7, 1.8)
Initial TMP dose, mg	760.0 (640.0, 960.0)	320.0 (320.0, 480.0)	320.0 (320.0, 320.0)	320.0 (320.0, 640.0)
Initial TMP dose, mg/kg	11.8 (11.3, 13.5)	6.5 (5.5, 7.9)	4.1 (3.7, 4.6)	6.0 (4.6, 10.0)
Creatinine, mg/dL	0.9 (0.8, 1.1)	0.8 (0.7, 0.9)	1.0 (0.9, 1.3)	0.9 (0.8, 1.1)
eGFR, mL/min/1.73 m^2^	74.1 (57.3, 93.1)	85.4 (70.0, 92.1)	70.3 (52.9, 83.6)	79.5 (59.2, 91.8)
Creatinine clearance, mL/min	48.1 (41.9, 84.5)	58.9 (43.6, 78.2)	59.3 (46.3, 74.2)	58.3 (43.3, 79.2)
Chronic kidney disease	8 (33.3%)	6 (16.2%)	11 (36.7%)	25 (27.5%)
Hospitalization	8 (33.3%)	10 (27.0%)	4 (13.3%)	22 (24.2%)
*N. farcinica*	4 (16.7%)	7 (18.9%)	6 (20.0%)	17 (18.7%)
Length of therapy, days^1^	178.5 (123.0, 226.0)	161.0 (94.5, 209.5)	146.0 (43.0, 184.0)	160.5 (93.5, 203.0)
Secondary prophylaxis^1^	3 (15.0%)	3 (11.4%)	6 (20.7%)	13 (15.5%)

Data are median (interquartile range) or number (percentage)

*Abbreviations* COPD, chronic obstructive pulmonary disease; CT, computed tomography; eGFR, estimated glomerular filtration rate; MRI, magnetic resonance imaging; TMP, trimethoprim

^1^*N*=84, after excluding 7 patients who did not complete primary therapy

**Fig. 2:**
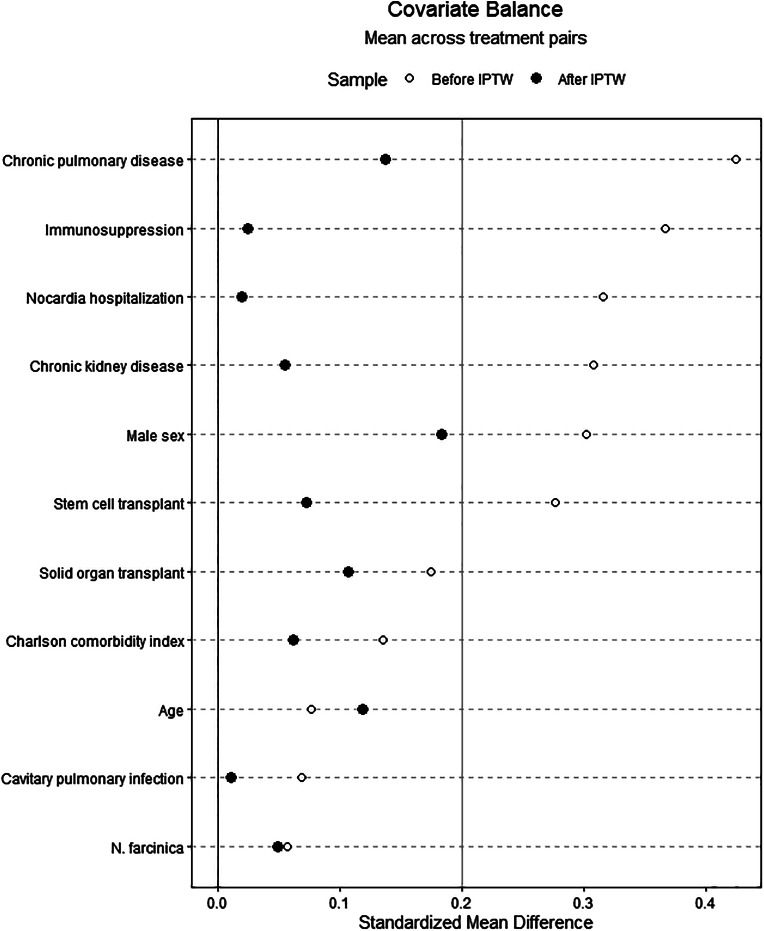
Love plot displaying averaged standardized mean differences of propensity score variables between trimethoprim-sulfamethoxazole dosing categories before and after inverse probability of treatment weighting (IPTW)

After calculating the propensity score and applying inverse probability of treatment weights, all included variables had an SMD ≤ 0.20 (Fig. 2). Notably, sex (SMD 0.182), chronic pulmonary disease (SMD 0.142), age (SMD 0.120), and history of solid organ transplantation (SMD 0.105) maintained the greatest differences between weighted groups with all other variables having an SMD < 0.10.

### One-year mortality and recurrence

**Fig. 3:**
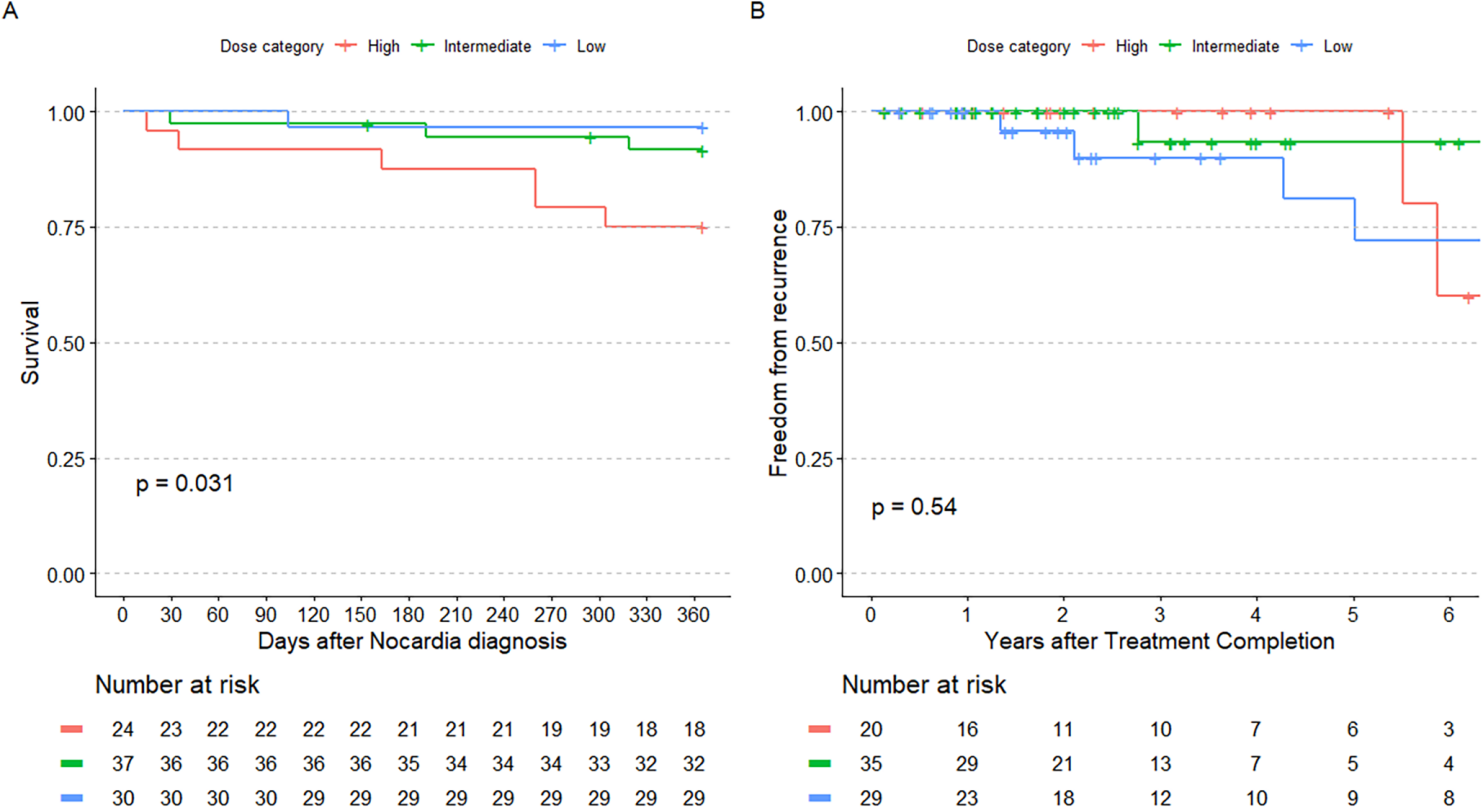
Kaplan-Meier curves illustrating the occurrence of one-year mortality (**A**) and all-time recurrence (**B**). The *p*-values were calculated via the log-rank test and the vertical hash marks represent censored patients

**Fig. 4:**
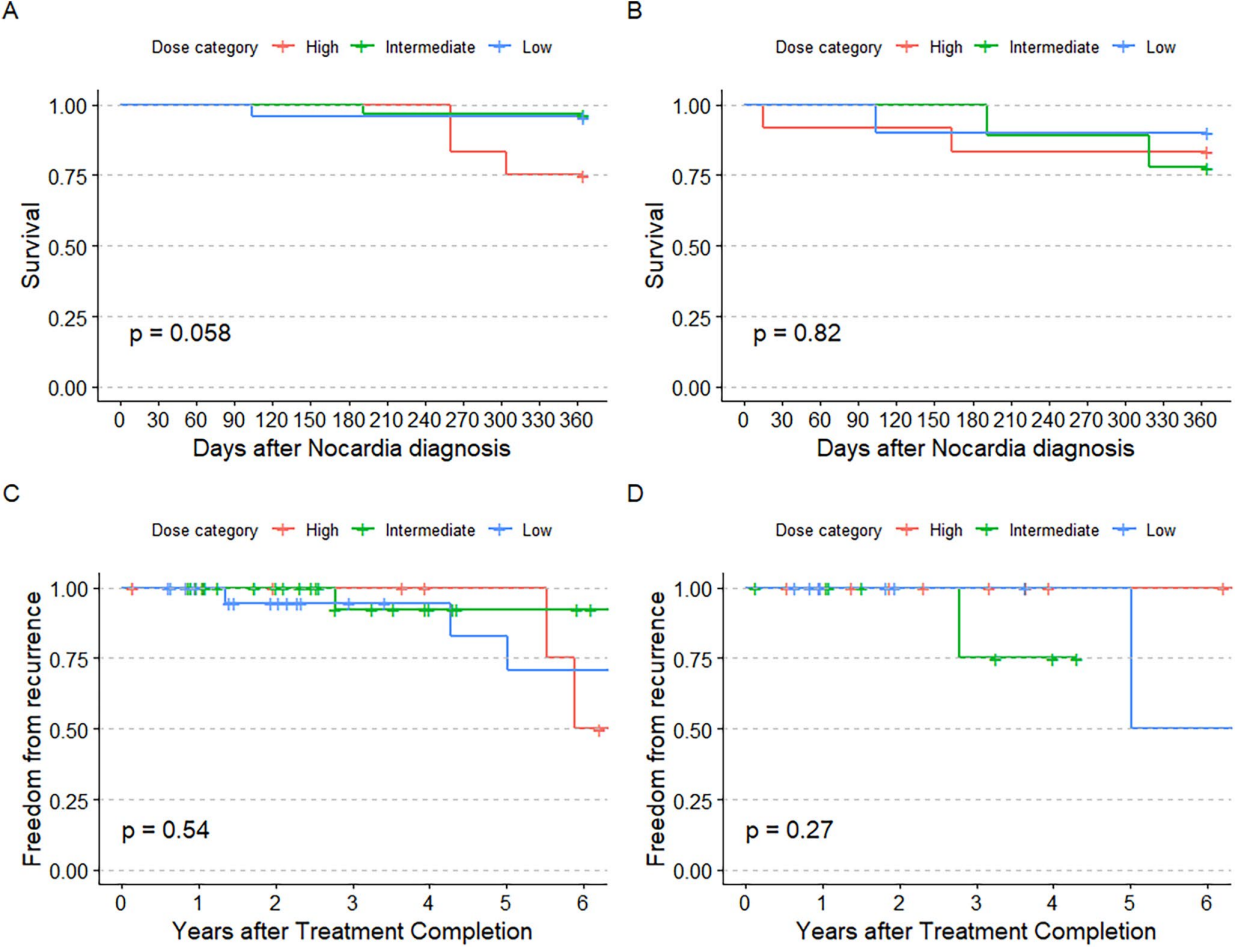
Kaplan-Meier curves illustrating one-year mortality in those with chronic pulmonary disease (**A**) and receiving immunocompromising medications (**B**), as well as all-time recurrence in those with chronic pulmonary disease (**C**) and receiving immunocompromising medications (**D**). The *p*-values were calculated via the log-rank test and the vertical hash marks represent censored patients

Ten patients (11.0%) died within 1 year of *Nocardia* infection diagnosis at a median time from diagnosis of 177.0 days (IQR 52.3, 260.0). Two deaths were directly attributable to nocardiosis. Two patients were lost to follow-up before 1 year after 154 and 295 days, respectively. Kaplan-Meier analysis showed a difference in one-year mortality between treatment groups (log-rank *p* = 0.031; Fig. 3A). After applying inverse probability of treatment weighting, those who received low-dose TMP-SMX dosing had a lower rate of one-year mortality than the high-dose group (hazard ratio [HR] 0.07, 95% confidence interval [CI] 0.01–0.68; *p* = 0.021). The intermediate-dose group had a lower, though non-significant, risk of one-year mortality compared to the high-dose group (HR 0.27, 95% CI 0.07–1.04; *p* = 0.057). In a sensitivity analysis comparing the high-dose group to all with a daily TMP dose < 10 mg/kg/day, lower dosed treatment was associated with lower mortality (HR 0.22, 95% CI 0.06–0.78; *p* = 0.018). In subgroup analyses, those with chronic pulmonary disease appeared to primarily account for the differences in mortality, though the difference was not statistically significant (log-rank *p* = 0.058, Fig. 4A-B).

After excluding 7 patients who did not complete primary therapy, 7 of 84 patients (8.3%) experienced *Nocardia* recurrence during follow-up, with a median time from completion of primary treatment of 490.0 days (116.0, 880.5). Three recurrences were with the same *Nocardia* species as the primary episode. Among those without recurrence, median follow-up after treatment completion was 490.0 days (IQR 116.0, 880.5). There was no difference in time-to-recurrence between treatment groups in Kaplan-Meier analysis (log-rank *p* = 0.540; Fig. 3B). After weighting, there remained no difference in risk of *Nocardia* recurrence among those with low-dose treatment (HR 1.22, 95% CI 0.25–5.99; *p* = 0.804) or intermediate-dose treatment (HR 0.36, 95% CI 0.07–1.81; *p* = 0.214) compared to a reference of high-dose TMP-SMX treatment. In a sensitivity analysis comparing the high-dose group to all with a daily TMP dose < 10 mg/kg/day, lower dosed treatment was not associated with a difference in recurrence rate (HR 0.52, 95% CI 0.11–2.56; *p* = 0.606). Results were also similar in the chronic pulmonary disease and immunosuppressing medication use subgroups (Fig. 4C-D).

### SMX serum concentrations

Nineteen patients had 38 peak SMX serum concentrations obtained during their treatment course. Of the 19 first concentrations per patient, the median SMX concentration was 100.0 µg/mL (IQR 85.0, 172.0), these were drawn a median 18.0 days (IQR 10.5, 28.5) since initiation of TMP-SMX therapy, and 7 (36.8%) resulted in a change of TMP-SMX dosing, with subsequent dose reduction in 6 of 7 patients. Eight patients had at least one subsequent SMX concentration drawn, which seldom resulted in a change in TMP-SMX dosing (Table 2).

**Table 2:** Timing, results, and response to sulfamethoxazole serum concentrations

Patient	Initial Dose	Level 1	Days	Response	Level 2	Days	Response	Level 3	Days	Response	Level 4	Days	Response	Level 5	Days	Response	One-year mortality	Recurrence
1	15.1	238	2	Reduce													No	No
2	4.9	105	4	Continue													No	No
3	4.1	94	9	Continue													No	No
4	3.8	57	9	Increase	156	16	Continue	132	28	Continue	145	70	Reduce	83	86	Continue	No	No
5	15.5	194	10	Reduce													**Yes**	--
6	15.3	100	11	Continue	57	59	Continue	60	87	Continue	40	148	Continue	66	192	Stop	No	No
7	3.7	85	14	Continue	99	21	Continue										No	No
8	14.3	174	15	Reduce													No	**Yes**
9	11.5	169	18	Reduce	117	34	Continue	55	46	Continue							No	No
10	12.7	93	18	Reduce	103	301	Continue										No	No
11	5.0	85	18	Continue	66	32	Continue	110	48	Continue	102	103	Continue				No	No
12	7.9	172	20	Continue													No	No
13	8.3	172	28	Continue	234	117	Continue	224	126	Reduce	153	138	Continue				No	No
14	12.7	228	28	Continue													No	No
15	18.8	55	29	Continue													No	No
16	7.3	127	38	Reduce	127	54	Continue										No	No
17	4.1	32	49	Continue													No	No
18	6.5	85	55	Continue													No	No
19	4.2	69	107	Continue													No	No

Initial dose is mg/kg/day of trimethoprim after accounting for baseline creatinine clearance

Days refers to the time after initiation of trimethoprim-sulfamethoxazole therapy

In unweighted analysis and incorporated as a time-dependent exposure, obtaining an SMX serum concentration was not associated with one-year mortality (HR 0.18, 95% CI 0.02–1.57; *p* = 0.121) or post-treatment recurrence (0.30, 95% CI 0.04–2.13; *p* = 0.228).

### Dose adjustments and early TMP-SMX discontinuation

Nineteen patients (20.9%) had their TMP-SMX dose adjusted 23 times. This involved reducing the dose 20 times and increasing 3 times. The reasons for dose reductions were adverse effects (*N* = 12) and response to SMX concentrations (*N* = 8), while reasons for dose increases included recommendation of an infectious diseases practitioner (*N* = 2) and response to SMX concentration (*N* = 1). One of the patients whose dose was increased later required reduction to their initial dose. Dose reductions were a median of 4.4 mg/kg/day (IQR 3.3, 5.2) and increases were median 2.8 mg/kg/day (IQR 2.8, 3.3). These dose adjustments resulted in a change in dosing category in 16 (69.6%) patients and occurred a median 23 days (IQR 14.0, 56.5) after treatment initiation.

Sixteen patients (17.6%) discontinued TMP-SMX early due to an adverse effect occurring at a median 38.5 days (20.8, 65.5) after start of therapy. The adverse effects resulting in discontinuation included elevated creatinine (*N* = 5; 31.2%), rash (*N* = 5, 31.2%), leukopenia (*N* = 2; 12.5%), nausea/vomiting (*N* = 2, 12.5%), elevated liver enzymes (*N* = 1, 6.2%), and edema (*N* = 1, 6.2%). Patients were transitioned to minocycline (*N* = 5, 31.2%), doxycycline (*N* = 4, 25.0%), amoxicillin-clavulanate (*N* = 2, 12.5%), moxifloxacin (*N* = 2, 12.5%), amoxicillin-clavulanate and moxifloxacin (*N* = 1, 6.2%), ceftriaxone (*N* = 1, 6.2%), or no therapy (*N* = 1, 6.2%). Among those who stopped TMP-SMX early, patients receiving high-dose therapy terminated TMP-SMX earlier (median 18.5 days versus 49.5 days in the intermediate-dose group and 47.0 days in the low-dose group) and included all with nausea/vomiting (*N* = 2) as their reported effect. One patient each experienced one-year mortality and post-treatment recurrence, respectively.

In total, 33 patients required dose adjustment or early TMP-SMX discontinuation. Patients initially receiving high-dose TMP-SMX had the highest rate of dose adjustment or early discontinuation (*N* = 16, 66.7%) compared to intermediate- (*N* = 9, 24.3%) or low-dose treatment (*N* = 8, 26.7%), which was statistically significant (Fisher’s exact test: *p* = 0.001). For the individual components of dose adjustment and early discontinuation, this included 12 (50.0%) and 6 (25.0%) in the high-dose group, 5 (13.5%) and 4 (10.8%) in the intermediate-dose group, and 2 (6.7%) and 6 (20.0%) in the low-dose group, respectively.

## Discussion

In this study, we assessed outcomes of adult patients with non-disseminated pulmonary nocardiosis who were initially treated with TMP-SMX monotherapy. Those receiving low- or intermediate-dose TMP-SMX had lower rates of one-year mortality compared to the high-dose group, though the latter comparison was not statistically significant. However, rates of post-treatment recurrence were similar despite initial TMP-SMX dosing regimen. The high-dose group also had higher rates of either dose adjustment or early discontinuation of TMP-SMX. Finally, obtaining a SMX serum concentration typically did not result in a change in TMP-SMX dosing; though, when it did, this was usually a reduction in dose.

While we had hypothesized patients may have had similar outcomes regardless of TMP-SMX dosing, the finding that those receiving high-dose treatment had greater risk for one-year mortality was surprising. However, there are several potential explanations for these findings. Many of TMP-SMX’s adverse effects are dose dependent and adverse effects may have contributed to outcomes, as adverse reactions have been associated with higher dose treatment in patients with *Pneumocystis* pneumonia [26]. It is also possible that patients receiving initial high-dose TMP-SMX were more often transitioned to less effective therapy when requiring a change in treatment, noting duration of TMP-SMX use has been associated with improved outcomes [6]. Finally, there remains the possibility of residual confounding despite propensity weighting. Patients receiving higher dose therapy may have either had more acute illness or more high-risk comorbid conditions. In subgroup analyses, we found that this effect of dosing categorization on outcomes was more pronounced among those with chronic pulmonary disease than those receiving immunosuppressing medications. These different populations have been shown to have different rates of other complications, such as dissemination [3, 27], and perhaps there is a differential effect of TMP-SMX dosing based on immunocompromised status. Future studies should consider examining the effect of treatment strategies within these different populations.

We did not find any differences in rates of post-treatment *Nocardia* recurrence based on TMP-SMX dosing. *Nocardia* recurrence is relatively rare, with prior studies estimating this to occur in approximately 5% of patients who complete primary treatment [28–31]. It has been suggested that duration of treatment may be impactful in preventing *Nocardia* recurrence, with patients who receive longer therapy tending to have lower rates of recurrence [6, 31]. This factor may be more impactful than the dosing of TMP-SMX therapy. Notably, our analysis did not necessarily account for duration of primary treatment, though the intermediate- and low-dose groups tended to have shorter treatment durations without incurring higher rates of recurrence.

About 20% of this cohort had a peak SMX concentration obtained during treatment. However, this typically did not result in a change in management, with only about one-third having a change in treatment after this test. It is notable that the change in dose was typically a reduction, and we did not find any difference in clinical outcomes based on measurement of SMX concentration. This may suggest that this test offers a safe strategy to evaluate for potential dose adjustments, noting that most patients who received high-dose TMP-SMX required either a dose adjustment or early discontinuation with transition to an alternative agent. Indeed, a recent study of patients with *Nocardia* brain abscess suggested obtaining a SMX concentration was associated with lower rates of adverse effects, possibly due to early dose reductions [9]. Also, a prior study analyzing peak SMX concentrations among patients receiving high-dose TMP-SMX for *Pneumocystis*, *Nocardia*, or *Stenotrophomonas* infections have shown obtaining a SMX concentration may not affect clinical outcomes [10]. Recognizing this test likely does not improve mortality or recurrence rates, it is reassuring that considering reductions in TMP-SMX dose based on this result likely does not adversely affect clinical outcomes. This test could be considered as a tool for de-escalation of TMP-SMX dosing, particularly among patients felt to be at higher risk for adverse effects. It should also be noted that obtaining a SMX concentration was a provider-dependent decision, and there may have been important factors that influenced the decision to obtain this test.

High-dose TMP-SMX therapy was associated with an increased risk of requiring dose adjustment or early discontinuation. While some of these outcomes are related to the practice of obtaining SMX concentrations and anticipating a higher dose likely leads to higher results, the most common reason for these changes in therapy were related to adverse effects. While we did not specifically measure adverse effects overall, we did include those that led to a change in management, which suggests many patients cannot tolerate high-dose TMP-SMX for a full duration of treatment.

There are several limitations worth noting in this study. It was performed retrospectively and there are likely inherent sources of bias that may have altered the findings. Most significantly, there is likely residual confounding by indication, as patients who received higher dosed TMP-SMX likely had more severe nocardiosis at presentation. While weighting on the propensity for specific dosing strategy was incorporated to reduce these differences, this approach will not address unmeasured sources of confounding. There were also relatively few events overall, which limited our power to detect differences. We also limited our cohort to those with non-disseminated disease who received initial TMP-SMX monotherapy to better isolate the effect of TMP-SMX itself. This specific subset of patients with nocardiosis likely had relatively mild *Nocardia* infection, highlighted by most being treated entirely in the outpatient setting. These findings may not necessarily apply to those receiving combination therapy or with disseminated, non-pulmonary, or more severe nocardiosis. Finally, not all patients underwent brain imaging, and it is possible some may have had occult central nervous system infection and were thus misclassified as non-disseminated.

In summary, this study found receipt of low-dose TMP-SMX was associated with lower risk of one-year mortality than those receiving high-dose therapy. This difference was primarily driven by patients with chronic pulmonary disease, which may represent a difference in infection phenotype compared to those with compromised immune status. While there is likely residual confounding by indication in this association, we at least did not find evidence to support a higher dosing strategy in this population. However, a prospective randomized controlled trial would be needed to definitively address the optimal dosing of TMP-SMX for pulmonary nocardiosis. Risk of recurrence was overall similar regardless of dosing strategy, even among the chronic pulmonary disease and immunocompromising medication use subgroups. Patients receiving high-dose TMP-SMX often required dose adjustments or transition to an alternative agent earlier in their treatment course, highlighting the low tolerance of this dosing regimen. Finally, peak SMX serum concentrations typically do not result in a change of dosing, though often are used to justify dose decreases without clearly harming clinical outcomes. These findings question the historical high-dose TMP-SMX therapy typically used for pulmonary nocardiosis and suggests that lower dose therapy should be considered after assessing other pertinent clinical factors.

## Data Availability

Data is available from the corresponding author upon reasonable request.

## Abbreviations

CI: Confidence interval
HR: Hazard ratio
IQR: Interquartile range
MGIT: Mycobacterial Growth Indicator Tubes
SMD: Standardized mean difference
TMP-SMX: Trimethoprim-sulfamethoxazole
